# Clinical signs in functional (psychogenic) gait disorders: a brief survey

**DOI:** 10.1186/s40734-016-0031-1

**Published:** 2016-02-12

**Authors:** Leonard L. Sokol, Alberto J. Espay

**Affiliations:** Department of Neurology, James J and Joan A Gardner Center for Parkinson’s disease and Movement Disorders, University of Cincinnati, Cincinnati, Ohio USA; Department of Neurology and Rehabilitation Medicine, University of Cincinnati Academic Health Center, 260 Stetson St., Suite 4300, Cincinnati, OH 45267-0525 USA

**Keywords:** Psychogenic gait, Functional gait, Clinical signs

## Abstract

Clinical signs are critical in ascertaining the functional nature of a gait disorder. Four signs of gait impairment have been documented in the course of examining patients with clinically definite functional (psychogenic) movement disorders: “huffing and puffing” during standing and walking, manipulation-resistance dorsiflexion of the first toe, fixed plantar flexion and inversion, and marked discrepancy between ambulation with and without swivel chair assistance. While large studies are needed to ascertain their prevalence, sensitivity, and specificity, the identification of these signs may help elevate the diagnostic certainty of functional gait disorders.

**Electronic supplementary material:**

The online version of this article (doi:10.1186/s40734-016-0031-1) contains supplementary material, which is available to authorized users.

## Background

Functional (psychogenic) gait disorders (FGDs) are defined by ambulatory dysfunction with features that are inconsistent and incongruous with organic gait disorders [1]. Although a functional movement disorder should never be considered a diagnosis of exclusion, the recognition of unique clinical signs serves to support such diagnosis, without considering additional neurological investigations [2]. The gait abnormalities tends to be variable during the course of a functional presentation [3], but various phenotypic characteristics of FGDs have been documented including limping of one leg with near normal ambulation of the other; walking hesitantly as if navigating on ice; swaying erratically of the upper body axis [4], and a long list of alternative incongruous gait phenotypes (Table 1) [5]. In this Commentary, we aim to go beyond these abnormal ambulatory patterns in order to concentrate on four previously documented signs that support the functional etiology for ambulatory impairments, even in the absence of other functional features or overt psychological comorbidities. These signs may assist in the differential diagnosis of a functional gait disorder, but are certainly not sufficient as standalone entities. We caution that these 4 signs have not undergone rigorous quantitative analyses save 1; nonetheless, there has been reputed success of their applications in the clinic, supportive of a functional disorder when other patterns and patient history support it, and thus are worthy of follow-up and discussion herein.

**Table 1:** Patterns, signs, and supportive features in functional gait disorders

Overall functional gait patterns	Incongruent functional gait signs	Supportive additional features
Excessive slowness	“Huffing and puffing”	Variable resistance of feet or leg to passive manipulation
Astasia-abasia	Fixed toe extension	
Knee buckling	Fixed plantar flexion/inversion	Associated incongruent neurological findings
Tightrope walking		
Trembling walking	Swivel chair signs	
Truncal jerking		

## Excessive demonstration of effort during gait: “Huffing and puffing” sign

**Fig. 1:**
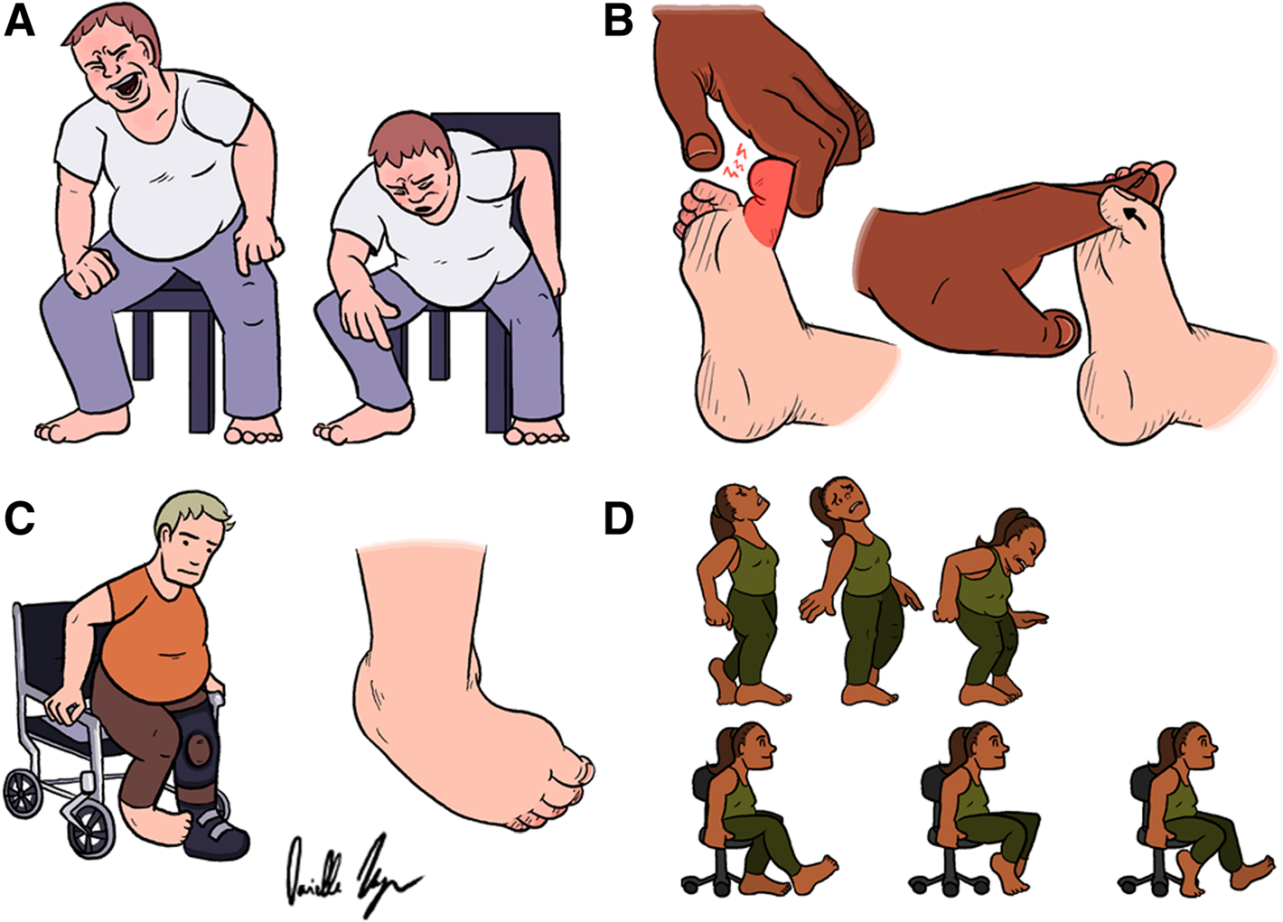
**a**. “Huffing and puffing” sign. Excessive demonstrations of effort are illustrated in this man attempting to rise from a chair, disproportionate to any strength or sensory deficits. **b**. Psychogenic toe sign. Left: the clinician attempts to flex the extended first toe with little success but substantial pain. Right: upon vigorous dorsiflexion of toes 2–5, spontaneous plantar flexion of the first toe is observed. **c**. Fixed plantar flexion sign. A fixed foot in plantar flexion and inversion prevents weight bearing, and forces the use of an assistive device (walker or wheelchair). A magnified view of the patient’s right foot is shown on the right. **d**. Swivel chair sign. Above, patient displays a bizarre gait pattern. Below, same patient is able to successfully propel herself in the swivel chair

The “huffing and puffing” sign (Fig. 1a, Additional file 1: Video 1), was coined after the observation that excessive demonstration of effort was often present in the context of ambulatory difficulty, discrepant with the extent of strength, balance, and postural impairments ascertained on neurological examination [6]. Such demonstrations of effort consist of grimacing, huffing, grunting, crying, and breath holding, among other effort-associated actions. These behaviors were evaluated between patients with FGD without associated pain (to avoid this confounding as source of effort) and age-, disease duration- and time-to-diagnosis-matched patients with organic gait disorder, cerebellar, spinocerebellar, and sensory ataxias. Despite greater severity of gait impairment in the organic gait disorder group, there was a larger magnitude of effort-related manifestations in the FGD group. While this sign exhibited low prevalence (sensitivity ranged from 17 – 57 % depending on pre-defined definitions, from most to least stringent), it was highly specific for FGD (specificity ranged from 89 – 100 %), raising the odds of such diagnosis by 13 times when present [6].

## Limited gait with incongruent dystonia (1/2): “Psychogenic toe” sign

The “psychogenic toe” sign (Fig. 1b, Additional file 2: Video 2), derives from a case study [7] of a 13-year-old male who exhibited a striatal-like toe on neurological examination within the context of clinically definite functional dystonia and weakness [7]. This sign is characterized by resistance to manipulation of an extended first toe, which can be forcibly flexed only at the expense of associated pain or by extending toes 2–5, which are held in tonic flexion. This sign needs to be distinguished from the spontaneous first-toe dorsiflexion seen in focal dystonias or dystonic manifestations of neurodegenerative disorders, including Parkinson disease and multiple system atrophy [8]. The organic striatal toe can be readily displaced with passive manipulation, without pain or resistance, and is not modified by passively extending toes 2–5 [7]. This phenotype is admittedly rare and may be restricted to the pediatric population, whereby dystonia tends to be a more common functional phenotype than tremor [9]. Indeed, our ongoing search in adults with this sign has been negligible in the years since this observation was published.

## Limited gait with incongruent dystonia (2/2): Fixed plantar flexion sign

Fixed plantar flexion and inversion of one foot or both feet, that appears suddenly or in rapid sequence, and which cannot be easily overcome with passive manipulation is typical of functional dystonia (Fig. 1c, Additional file 3: Video 3). Although dystonia is the primary phenotype, gait is an immediate casualty. Patients may be able to take a few limping steps while carefully guarding the affected foot by minimizing its weight bearing, when only one limb is affected (wheelchair-bound state is the rule among those with bilateral or severe unilateral leg involvement). In one large study, about 20 % of these patients may also carry the diagnosis of complex regional pain syndrome type 1 (formerly, reflex sympathetic dystrophy), reflecting secondary skin dysautonomia due to limb immobility [10]. This pattern of gait impairment has been well documented among soldiers with “shell shock” or “war neuroses” during World War I and other military conflicts [11].

## Incongruent ambulation: Swivel chair sign

The swivel chair test (Fig. 1d, Additional file 4: Video 4) may afford a means to evaluating the inconsistency of gait by way of comparing two mediums of ambulation [12]. Major gait discrepancies in gait performance between upright versus chair propelling walking has been suggested as an important sign in FGD. Inspired by an earlier observation by Charcot-trained Paul Oscar Blocq (1860 –1896), at the Salpêtrière, Michael Okun and his group formally compared the differential gait performance in patients with FGD before and after asking them to use a swivel chair to propel themselves forward and backward [12]. Compared with no changes in a control group of 9 consecutive organic gait impairment (7 of whom had Parkinson disease) 8/9 patients with FGD, who exhibited a range of bizarre gait patterns at baseline, were able to propel themselves on a swivel chair. Caveats regarding this observation are that the response to a swivel chair has not been examined in other organic gait disorders beyond neurodegenerative parkinsonisms and further studies will be needed to quantify the sign’s prevalence and related clinimetric properties. Also, in the absence of data from ataxic patients (where the sign may conceivably be positive), the reader is cautioned about the likelihood of misclassification when relying on this sign disproportionate to other neurological examination features.

## Conclusions

The bizarre nature of certain gait abnormalities, alone, does not suffice to diagnose a presentation as FGD, a pitfall illustrated by the unusual gait of patients with chorea-acanthocytosis and “limp man syndrome” [13]. Identifying previously reported clinical signs, despite the need to further validate their relevance in FGD, could be helpful in ascertaining the diagnosis while explaining the futility of unnecessary additional laboratory evaluations. A clinically definite diagnosis of FGD also serves to steer therapeutic efforts away from pharmacotherapy, and the associated iatrogenic harm, and toward multidisciplinary physical and cognitive behavioral therapies.

## Consent

Consent for publication of videos was obtained from each of the patients. Patients signed a standard institutional consent form, which includes publication in medical journals.

## Additional files

Additional file 1: Video 1. Excessive demonstration of effort with groaning (“huffing and puffing” sign) in this patient with associated functional parkinsonism and dystonia. (M4V 5063 kb) Additional file 2: Video 2. Fixed dorsiflexed toe interfering with gait, and mimicking a striatal toe. The dorsiflexion is resistant to passive manipulation but spontaneously resolves with forced dorsiflexion of the other toes. (M4V 10296 kb) Additional file 3: Video 3. Limited gait given fixed dystonia, rendering the leg unable to bear weight. (M4V 9507 kb) Additional file 4: Video 4. Limited gait on a cane is partly overcome through the use of a swivel chair. (M4V 15491 kb)

## Abbreviations

FGD: Functional (psychogenic) gait disorders
